# Impact of extending the original criteria in the Chemoradiotherapy for Oesophageal Cancer followed by Surgery Study (CROSS) regimen on treatment outcome in locally advanced esophageal cancer patients

**DOI:** 10.1016/j.esmoop.2025.105098

**Published:** 2025-05-15

**Authors:** H.H. Wang, I.M. Nolte, R.H.A. Verhoeven, V. Oppedijk, B. van Etten, G. Kats-Ugurlu, J.T.M. Plukker, G.A.P. Hospers

**Affiliations:** 1 Department of Medical Oncology, University of Groningen, University Medical Center Groningen, Groningen, The Netherlands; 2 Department of Epidemiology, University of Groningen, University Medical Center Groningen, Groningen, The Netherlands; 3 Department of Research & Development, Netherlands Comprehensive Cancer Organisation (IKNL), Utrecht, The Netherlands; 4 Department of Medical Oncology, Amsterdam UMC, location University of Amsterdam, Amsterdam, The Netherlands; 5 Cancer Center Amsterdam, Cancer Treatment and Quality of Life, Amsterdam, The Netherlands; 6 Department of Radiation Oncology, Radiotherapeutic Institute Friesland, Leeuwarden, The Netherlands; 7 Department of Surgical Oncology, The Netherlands; 8 Department of Pathology, University of Groningen, University Medical Center Groningen, Groningen, The Netherlands

**Keywords:** esophageal carcinoma, neoadjuvant chemoradiotherapy, surgery, treatment outcome, survival, pathological response

## Abstract

**Background:**

The Chemoradiotherapy for Oesophageal Cancer followed by Surgery Study (CROSS) regimen is currently offered to locally advanced esophageal cancer patients beyond the original eligibility criteria. This national population-based study assessed the safety in implementation regarding treatment outcome when extending these criteria.

**Patients and methods:**

Locally advanced esophageal cancer (cT1N+/T2-4aN0-3/M0) patients (*n* = 5061) from the Netherlands Cancer Registry treated according to the neoadjuvant chemoradiotherapy (nCRT) CROSS regimen between 2015 and 2022 were analyzed. A total of 1958 complied with the original criteria (O-CROSS group) and 1348 with one or more extended criteria (tumor length >8 cm, age >75 years, WHO score >2 and/or weight loss >10%) (E-CROSS group), eventually followed by resection in 1342 O-CROSS patients and 852 E-CROSS patients. Primary outcome was overall survival (OS), i.e. time interval from onset of nCRT (OS-nCRT) and from date of surgery (OS-surgery) until death or last follow-up. Secondary outcomes were disease-free survival, pathological complete response (pCR), surgical radicality, post-operative morbidity and mortality. Data were analyzed using the Kaplan–Meier method and Cox proportional hazards models.

**Results:**

OS-nCRT was significantly lower in the E-CROSS compared with the O-CROSS (median of 30.3 months, 95% confidence interval 27.2-33.5 months versus 45.9 months, 95% CI 38.4-53.4 months, *P* < 0.001). Similarly, differences were observed in OS-surgery. When OS-nCRT and OS-surgery were adjusted for baseline covariates, however, no difference was found between both groups. Moreover, no differences were observed in disease-free survival, surgical radicality, and pCR. While not affecting post-operative mortality, significantly more anastomotic leakages and thromboembolic post-operative complications were seen in the O-CROSS group.

**Conclusion:**

Extending the CROSS criteria was associated with lower OS, which was caused by the higher age, weight loss >10% and WHO score in the E-CROSS group. The CROSS regimen can be used in a ‘real-world’ setting but individual factors that may contribute to OS should be considered in decision-making.

## Highlights

• Extending the criteria had no significant impact on DFS, pCR, surgical radicality, and post-operative mortality.
• Extending the original CROSS criteria was associated with a lower OS.
• The lower OS in the E-CROSS was caused by higher age, weight loss >10%, and higher WHO performance score.
• Present enlargement of the original CROSS criteria can be implemented safely in practice.
• Individual factors that may contribute to OS should be considered in decision-making.

## Introduction

During the analysis the Chemoradiotherapy for Oesophageal Cancer followed by Surgery Study (CROSS) regimen is still standard treatment in potentially resectable locally advanced esophageal cancer (EC); [clinical TNM (tumor–node–metastasis) classification cT1N+/T2-4aNany/M0] in the Netherlands.^1^ The Dutch randomized ‘CROSS’ trial applied strict inclusion criteria, but based on significant long-term survival data, this regimen has also been administered in daily clinical practice to patients with a curative resectable tumor beyond the original (O-CROSS) criteria.^1^ Our previous small-scale study (*n* = 161) had shown that patients who did not meet the O-CROSS criteria had a worse overall survival (OS) and disease-free survival (DFS).^2^ Large-scale data on OS in patients treated according to the CROSS regimen in a real-world setting, however, are still lacking. In this national cohort study, the effect of extending the CROSS inclusion criteria on both OS and DFS in EC patients was assessed. It was hypothesized that there would be no difference in OS between patients in the O-CROSS and E-CROSS.

## Methods

### Patients

This retrospective national cohort study was conducted with approval of the local Ethical Board (registration number 201900821). Data were obtained from the Netherlands Cancer Registry, registering data on cancer diagnoses in the Netherlands. Our database consisted of all relevant EC patient-related information [i.e. age, sex, weight, length, and pre-treatment (cardiac, pulmonary, renal, hepatic, infection) comorbidities], tumor-related data (i.e. biopsy proven histopathological EC diagnosis, pre-treatment cT and cN stage, tumor length), surgical treatment-related data (i.e. type of surgery, surgical complications), and pathology data (presence/absence of earlier histologically proven EC in the resection specimen after neoadjuvant chemoradiotherapy (nCRT) with no, partial or complete pathological response (pCR) including ypT and ypN stage, tumor differentiation, and tumor-free resection margins >1 mm both at length and circumferentially according to the Royal College of Pathologists^3^). In total, 5061 EC patients with esophageal adenocarcinoma (EAC) or esophageal squamous cell carcinoma (ESCC) treated between 2015 and 2022 according to the CROSS regimen were eligible for inclusion. The CROSS regimen consisted of five weekly treatments of carboplatin (area under the curve = 2) and paclitaxel (50 mg/m^2^) with concurrent radiotherapy (total dose of 41.4 Gy in daily fractions of 1.8 Gy) followed by surgical resection after nCRT.

### Patient selection

The O-CROSS group (*n* = 1958) consisted of patients who met the original criteria [i.e. tumor length ≤8 cm, locally advanced EC (clinical TNM: cTX-1N+/T2-4aNany/M0), age ≤75 years, WHO performance score ≤2, weight loss ≤10%]. The E-CROSS group (*n* = 1348) contained patients who met one or more of the extended criteria, including tumor length >8 cm, age >75 years, WHO score >2 and/or weight loss >10%. Due to missing data regarding one or more of the E-CROSS criteria, a total of 1755 patients could not be categorized into either the O-CROSS group or E-CROSS group and were therefore considered as the ‘undefined group’. Patients with a cTx were included, as it was assumed that these patients had a cT below cT4b, since their tumors were regarded as resectable at time of diagnosis. In selecting patients for a curative intended treatment during the original CROSS study, the 6th edition of the UICC TNM staging was applied. With the introduction of the 7th and 8th UICC editions, however, cT4a tumors are included as these tumors are resectable and treated accordingly.

**Figure 1:**
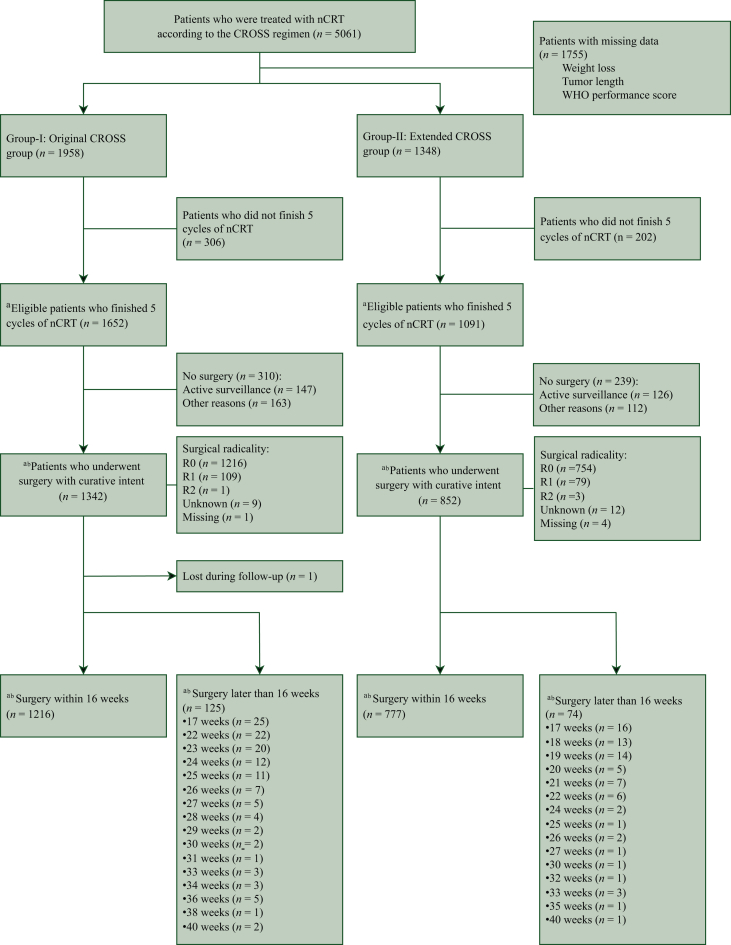
**CONSORT flow diagram of patients included in the original versus extended CROSS group.** CROSS, Chemoradiotherapy for Oesophageal Cancer followed by Surgery Study; nCRT, neoadjuvant chemoradiotherapy. ^a^Included in the nCRT analyses. ^b^Included in the surgical analyses.

Data regarding other CROSS criteria were identical or not available from the Netherlands Cancer Registry and therefore not evaluated. Only patients who finished the complete nCRT were included in the analyses. So, the study group consisted of 1652 patients in the O-CROSS and 1091 patients in E-CROSS. The O-CROSS and E-CROSS included patients who underwent a curative intended radical surgical resection [O-CROSS *n* = 1342 (81.2%) versus E-CROSS *n* = 852 (78.1%)] and patients who ultimately refrained from or did not undergo surgery after complete nCRT [O-CROSS *n* = 310 (18.8%) versus E-CROSS *n* = 239 (21.9%)]. To differentiate between patients who underwent surgery directly after completion of nCRT and patients who underwent surgery due to a wait-and-see strategy, a distinction was made between patients who underwent surgery ≤16 weeks (O-CROSS *n* = 1216 versus E-CROSS *n* = 777) and >16 weeks after completion of nCRT (O-CROSS *n* = 125 versus E-CROSS *n* = 74) (Figure 1).

### Staging and restaging

Clinical staging consisted of endoscopy, computed tomography combined with [^18^F]2-fluoro-2-deoxy-D-glucose–positron emission tomography and endoscopy ultrasound scan. Patients were staged according to the Union for International Cancer Control (UICC) TNM Classification according to the 7th edition (period 2015-2017) and according to the 8th UICC edition after 2017. As there are no substantial changes in the definitions of the TNM categories both are comparable, with renaming stage subgroups and gastroesophageal adenocarcinoma growing >2 cm distally.^3^, ^4^, ^5^

### Pathology

Pathological assessment of the resected specimen was carried out according to the approved local protocol in the respective medical centers. The histopathological tumor type, local tumor extension within the esophagus, involvement of locoregional lymph nodes, and resection margins were evaluated and reported by experienced upper gastrointestinal pathologists.

### Follow-up

Follow-up data included tumor recurrence and death according to the local institutional protocol. Tumor recurrence was evaluated either radiologically on clinical evidence and/or proven cyto/histopathological biopsies. It should be noted that data on tumor recurrences were only completely available in the EC patients from 2015 until 2017 by the Netherlands Cancer Registry.

### Outcome measurements

The primary outcome was OS, defined as the time interval between start of nCRT until date of death or last day of follow-up based on all cases (OS-nCRT). For patients who underwent surgery, the OS was also assessed accordingly using the time interval from the date of surgery (OS-surgery).

Secondary outcomes were DFS, defined as time of start nCRT or date of surgery (DFS-nCRT or DFS-surgery, respectively) until date of clinical or pathological tumor recurrence; pCR (ypT0N0 and ypT0); curative radical resection [no microscopic residual tumor at the circumferential margin: R0 (CRM neg. >1 mm) versus microscopic irradical: R1 (CRM ≤1 mm) versus macroscopic irradical/residual tumor: R2]; post-operative morbidity (complications within 30 days after surgery) and mortality (≤30 and ≤90 days after surgery). Patients with R2 resected tumors (*n* = 4) were excluded from the DFS analyses, as they are inherently not disease free. From 2015 until 2019 (*n* = 1457), post-operative complications were registered per individual category (i.e. pulmonary, cardiac). From 2020 onwards (*n* = 737), the Netherlands Cancer Registry categorized post-operative complications solely as requiring surgical, endoscopic, or radiological intervention or complications leading to re-admission to the intensive care unit. Therefore, two categories of post-operative complications (before and after 2020) were included in the analyses. Lastly, separate analyses were conducted for each histological subtype (EAC, ESCC).

### Statistics

Chi-square tests were used comparing O-CROSS and E-CROSS to describe differences in categorical variables. The Mann–Whitney *U*-test was used for non-normally distributed variables. For the pathological and post-operative characteristics, the group of patients who underwent surgery were first analyzed and thereafter the subgroups of patients who had surgery within or beyond 16 weeks after nCRT.

Kaplan–Meier curves were used to display OS and DFS. Cox proportional hazard analyses were carried out to assess prognostic variables of individual parameters. Outcomes with a *P* value <0.10 for the univariable analyses were included in a multivariable analysis. To assess whether a variable showed a significantly different effect on OS in the E-CROSS compared with the O-CROSS group, an interaction term was added of that variable with the CROSS variable. A *P* value <0.05 was considered as statistically significant. Statistical analyses were carried out with IBM SPSS statistics for Windows, Version 23.0 (IBM Corp., Armonk, NY) and RStudio Desktop for Windows 10+, Version 2024.4.1.748 (Posit, PBC, Boston, MA).

## Results

### Patient characteristics

Patient characteristics are summarized in Table 1. There was a significant difference in sex between the O-CROSS and E-CROSS groups (79.5% versus 75.9% males, respectively, *P* = 0.027). In line with the inclusion criteria, E-CROSS patients were significantly older (69 years versus 66 years, respectively, *P* < 0.001), had a higher WHO performance score (*P* < 0.001) and a greater tumor length (6.0 cm versus 4.0 cm, respectively, *P* < 0.001) compared with the O-CROSS group. Moreover, E-CROSS patients were more likely to have a higher cTNM stage (*P* = 0.001). The E-CROSS group had a significantly higher number of comorbidities, including myocardial infarction (7.2% versus 5.1%, *P* = 0.025), diabetes mellitus (17.4% versus 13.9%, *P* = 0.012), and mild liver disorders (1.1% versus 0.4%, *P* = 0.039). In addition, the follow-up period for the E-CROSS subgroup was shorter with a median follow-up of 21.7 months [interquartile range (IQR) 12.4-42.8 months] compared with 26.8 months (IQR 10.7-45.0 months) for O-CROSS (*P* < 0.001).

**Table 1:** Patient and tumor characteristics of the complete original and extended CROSS groups

	Original CROSS (*N* = 1652), *n* (%)	Extended CROSS (*N* = 1091), *n* (%)	*P* value^a^
Sex, male	1313 (79.5)	828 (75.9)	*0.027*
Age, years, median (IQR)^b^	66 (60-71)	69 (61-76)	*<0.001*
Weight loss (%), (median, IQR)^b^	2.6 (0.0-5.6)	11.3 (5.2-14.7)	*<0.001*
Histology			0.175
Adenocarcinoma	1324 (80.1)	897 (82.2)	
Squamous cell carcinoma	328 (19.9)	194 (17.8)	
Tumor location			0.264
Proximal	11 (0.7)	6 (0.5)	
Middle	183 (11.1)	131 (12.0)	
Distal	1433 (86.7)	932 (85.4)	
Overlapping	8 (0.5)	13 (1.2)	
Not specified	17 (1.0)	9 (0.8)	
WHO performance score			*<0.001*
0	1008 (61.0)	509 (46.7)	
1	612 (37.0)	477 (43.7)	
2	32 (1.9)	47 (4.3)	
3	0 (0.0)	3 (0.3)	
4	0 (0.0)	1 (0.1)	
Unknown	0 (0.0)	54 (4.9)	
Missing	0 (0.0)	0 (0.0)	
Tumor length, cm, median (IQR)^b^	4.0 (3.0-6.0)	6.0 (4.0-9.0)	*<0.001*
Missing	0 (0.0)	88 (8.1)	
Clinical TNM stadium			*0.001*
1	4 (0.2)	2 (0.2)	
1A	1 (0.1)	0 (0.0)	
1B	60 (3.6)	33 (3.0)	
2	116 (7.0)	47 (4.3)	
2A	84 (5.1)	57 (5.2)	
2B	209 (12.7)	102 (9.3)	
3	789 (47.8)	521 (47.8)	
3A	103 (6.2)	71 (6.5)	
3B	54 (3.3)	51 (4.7)	
3C	11 (0.7)	13 (1.2)	
4A	202 (12.2)	179 (16.4)	
X	19 (1.2)	15 (1.4)	
**Comorbidities**			
Myocardial infarct	85 (5.1)	79 (7.2)	*0.025*
Perivascular disease	94 (5.7)	71 (6.5)	0.381
COPD	221 (13.4)	167 (15.3)	0.158
Diabetes mellitus	229 (13.9)	190 (17.4)	*0.012*
Renal disease	32 (1.9)	28 (2.6)	0.275
Mild liver disorders	7 (0.4)	12 (1.1)	*0.039*
Follow-up, months, median (IQR)^b^	26.8 (10.7-45.0)	21.7 (12.4-42.8)	*<0.001*
Missing	1 (0.1)	0 (0.0)	

The italic values indicate statistical significance at *P* < 0.05.

CROSS, Chemoradiotherapy for Oesophageal Cancer followed by Surgery Study; IQR, interquartile range; TNM, tumor–node–metastasis.

a Mann–Whitney *U* test.

b Likelihood ratio test.

### Overall survival

**Figure 2:**
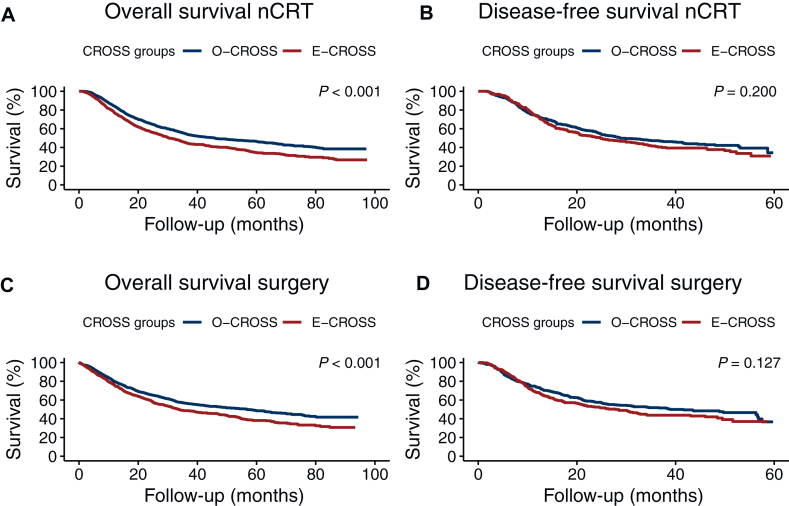
The overall and disease-free survival in the original (O-CROSS) versus extended (E-CROSS) CROSS group, from first date of neoadjuvant chemoradiotherapy (nCRT) (A, B); from date of surgery (C, D). CROSS, Chemoradiotherapy for Oesophageal Cancer followed by Surgery Study.

**Supplementary Fig S1:**
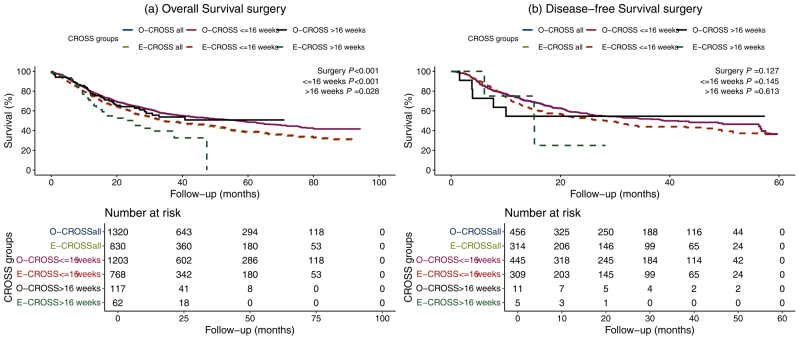
**Surgical subanalyses of patients who underwent surgery, within16weeks and16sixteen weeks**. Overall survival (a); and disease-free survival (b) in the O-CROSS vs. E-CROSS group.

Figure 2 depicts the Kaplan–Meier curves illustrating both OS and DFS for O-CROSS and E-CROSS. The OS-nCRT differed significantly between the two groups (*P* < 0.001; Figure 2A), with a median of 45.9 months (95% CI 38.4-53.4 months) in the O-CROSS and 30.3 months (95% CI 27.2-33.5 months) in the E-CROSS group. Similarly, the OS-surgery displayed a significant difference between both groups (*P* < 0.001; Figure 2C), with a median of 56.8 months (95% CI 46.6-67.0 months) in the O-CROSS group and 33.6 months (95% CI 26.6-40.7 months) in the E-CROSS group. These differences persisted in patients who underwent surgery within and beyond 16 weeks after nCRT (Supplementary Figure S1, available at https://doi.org/10.1016/j.esmoop.2025.105098).

Supplementary Tables S1 and S2, available at https://doi.org/10.1016/j.esmoop.2025.105098, display the survival results of the E-CROSS criteria and CROSS subgroups. Upon univariable Cox regression for OS-nCRT, sex, the CROSS variable, age, WHO performance status, squamous cell carcinoma, high-grade differentiation, cTNM, ypT stage, ypN stage, surgical radicality, tumor regression grade, comorbidities (perivascular disease and renal disease) and post-operative complication (anastomotic leakage) had a *P* value <0.10 in the univariable analyses with OS-nCRT. The impact of each included CROSS criterium on OS is shown in the forest plot (Supplementary Figure S2, available at https://doi.org/10.1016/j.esmoop.2025.105098). Independent prognostic factors for OS-nCRT in the multivariable Cox regression analysis were high-grade differentiation [hazard ratio (HR) 1.34, 95% CI 1.24-1.65, *P* < 0.001], ypN stage (*P* < 0.001), surgical resection (*P* < 0.001), pathological response (*P* = 0.049), and anastomotic leakage (HR 1.22, 95% CI 1.00-1.49, *P* = 0.049). The CROSS variable was not significant anymore (*P* = 0.286). Similar independent prognostic factors for OS-surgery were found in the multivariable Cox regression analyses without anastomotic leakage, but with WHO performance score (*P* = 0.041) and pulmonary post-operative complication (HR 1.28, 95% CI 1.09-1.51, *P* = 0.003) being significant. The presence of comorbidity perivascular disease was significant in the E-CROSS subgroup analysis (HR 0.52, 95% CI 0.28-0.97, *P* = 0.040) but not in the overall cohort, suggesting that the impact of perivascular disease on OS differs between both groups.

**Figure 3:**
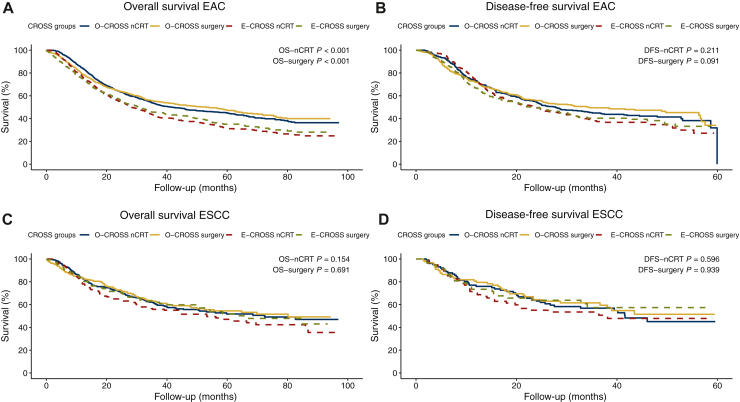
Separate histopathological analysis of the overall and disease-free survival in the original (O-CROSS) versus extended (E-CROSS) CROSS group, from first date of neoadjuvant chemoradiotherapy (nCRT) and from date of surgery in esophageal adenocarcinoma (EAC) (A, B); in esophageal squamous cell carcinoma (ESCC) (C, D). CROSS, Chemoradiotherapy for Oesophageal Cancer followed by Surgery Study; DFS, disease-free survival.

A separate analysis of histological subtypes showed that both the OS-nCRT and OS-surgery of EAC differed significantly between the O-CROSS and E-CROSS groups, respectively (both *P* < 0.001; Figure 3A), but not within the ESCC subtype (*P* = 0.154; *P* = 0.691; Figure 3C). For OS-nCRT, the O-CROSS group with EAC showed a median OS of 41.6 months (95% CI 33.8-49.4 months), while the E-CROSS group had a median OS of 28.4 months (95% CI 24.9-31.8 months). Following surgery, the EAC O-CROSS group had a median OS of 53.9 months (95% CI 43.8-64.0 months) compared with a median of 31.2 months (95% CI 25.6-36.9 months) for the E-CROSS group.

### Disease-free survival

The DFS-nCRT did not differ between the two subgroups (*P* = 0.200; Figure 2B), with a median of 28.9 months (95% CI 21.1-36.7 months) and 24.8 months (95% CI 18.5-31.0 months) in the O-CROSS and E-CROSS groups, respectively. Similarly, the DFS-surgery did not differ significantly (*P* = 0.127; Figure 2D), with a median of 38.9 months (95% CI 25.0-52.9 months) and 27.9 months (95% CI 21.0-34.7 months) for the O-CROSS and E-CROSS groups, respectively. Upon stratifying patients based on timing of surgery ≤ or >16 weeks after nCRT, no significant differences were found either (Supplementary Figure S1, available at https://doi.org/10.1016/j.esmoop.2025.105098). Upon univariable Cox regression for DFS-nCRT, high-grade differentiation, cTNM, ypN stage, surgical resection, and tumor regression grade had a *P* value <0.10 in the univariable analysis. The impact of each included CROSS criterium on DFS is shown in the forest plot (Supplementary Figure S2, available at https://doi.org/10.1016/j.esmoop.2025.105098). Independent prognostic factors for DFS-nCRT in the multivariable Cox regression analysis were high-grade differentiation (HR 1.49, 95% CI 1.19-1.87, *P* < 0.001], ypN stage (*P* < 0.001) and pathological response (*P* < 0.001) (Supplementary Table S3, available at https://doi.org/10.1016/j.esmoop.2025.105098). The multivariable Cox regression analysis for DFS-surgery showed similar results, except the clinical TNM stage being a significant prognostic factor (*P* = 0.037) (Supplementary Table S4, available at https://doi.org/10.1016/j.esmoop.2025.105098). In neither multivariable analysis was the CROSS variable significant (*P* = 0.449 and *P* = 0.206, respectively).

Separate analyses of the histological subtypes showed no differences in DFS-nCRT in the EAC and ESCC subtypes between both O-CROSS and E-CROSS (Figure 3B and D).

### Pathological complete response

Table 2 depicts the pathological characteristics of the O-CROSS and E-CROSS patients. A significant higher ypT stage was observed in patients from E-CROSS (*P* = 0.001, Table 2). No significant differences, however, were observed in pCR (ypT0N0; ypT0). In total, 316 patients (23.5%) with a curative resection in the O-CROSS group achieved a total pCR (ypT0N0), compared with 161 patients (18.9%) in E-CROSS group, which was not significant (*P* = 0.065). Also, no significant difference was found in in achieving a local pCR (ypT0) between the O-CROSS group (353 patients; 26.4%) and E-CROSS group (198 patients; 23.2%). Separate analyses for EAC showed that significantly more O-CROSS patients reached a local pCR compared with E-CROSS (20.3% versus 18.9%, respectively, *P* = 0.006). No differences were found in pathological response between both groups in the ESCC subtype (Supplementary Table S5, available at https://doi.org/10.1016/j.esmoop.2025.105098).

**Table 2:** Pathological tumor characteristics in the original and extended CROSS groups after curative resection

	Original CROSS (*N* = 1342), *n* (%)	Extended CROSS (*N* = 852), *n* (%)	*P* value^a^
pCR (ypT0N0)	316 (23.5)	161 (18.9)	0.065
pCR (ypT0)	353 (26.4)	198 (23.2)	0.197
ypT-stage			*0.001*
Tx	12 (0.9)	7 (0.8)	
T0	350 (26.1)	196 (23.0)	
Tis	1 (0.1)	0 (0.0)	
T1a	48 (3.6)	28 (3.3)	
T1b	192 (14.3)	95 (11.2)	
T2	269 (20.0)	157 (18.4)	
T3	465 (34.6)	355 (41.7)	
T4a	5 (0.4)	9 (1.1)	
T4b	0 (0.0)	4 (0.5)	
ypN stage			0.217
N0	844 (62.9)	497 (58.3)	
N1	295 (22.0)	210 (24.6)	
N2	135 (10.1)	98 (11.5)	
N3	63 (4.7)	42 (4.9)	
Missing	5 (0.4)	5 (0.6)	
Pathologic differentiation			0.158
Low grade	685 (51.0)	406 (47.7)	
High grade	455 (33.9)	323 (37.9)	
Unknown	202 (15.1)	123 (14.4)	
Resection			0.285
R0	1216 (90.6)	754 (88.5)	
R1	109 (8.1)	79 (9.3)	
R2	1 (0.1)	3 (0.4)	
Unknown	14 (1.0)	12 (1.4)	
Missing	2 (0.2)	4 (0.5)	

The italic values indicate statistical significance at *P* < 0.05.

CROSS, Chemoradiotherapy for Oesophageal Cancer followed by Surgery Study; pCR, pathologic complete response; R0, microscopically radical resection; R1, microscopically irradical resection margin; R2, locoregional tumor residue; ypN, pathologic node stage; ypT, pathologic tumor stage.

a Likelihood ratio test.

### Surgical radicality

The proportion of patients with a curative radical resection (R0) was not significantly different between the O-CROSS and E-CROSS groups (90.6% versus 88.5%, *P* = 0.285; Table 2). Histological subtype analyses showed a significant difference in surgical radicality between O-CROSS and E-CROSS in ESCC (92.1% versus 90.4%, respectively, *P* = 0.047; Supplementary Table S5, available at https://doi.org/10.1016/j.esmoop.2025.105098).

### Post-operative morbidity and mortality

Significant differences in anastomotic leakage (O-CROSS versus E-CROSS, 21.4% versus 17.1%, *P* = 0.043) and thromboembolic post-operative complications (O-CROSS versus E-CROSS, 2.9% versus 1.2%, *P* = 0.027) were observed in the period 2015-2019. No significant difference in post-operative morbidity was observed between the O-CROSS and E-CROSS groups in the period 2020-2022 (*P* = 0.516, Table 3).

**Table 3:** Post-operative complications in patients who underwent surgery

	O-CROSS (*N* = 1342), *n* (%)	E-CROSS (*N* = 852), *n* (%)	*P* value^a^
Post-operative mortality			0.774
<30 Days	24 (1.8)	19 (2.2)	
<90 Days	40 (3.0)	38 (4.5)	
Post-operative complications (2015-2019)	(*n* = 889)	(*n* = 568)	
Chyle leak	95 (10.7)	48 (8.5)	0.158
Complication regarding recurrent nerve	30 (3.4)	16 (2.8)	0.550
Wound abscess/infection	133 (15.0)	67 (11.8)	0.084
Anastomotic leakage	190 (21.4)	97 (17.1)	*0.043*
Pulmonary	290 (32.6)	185 (32.6)	0.984
Cardiac	137 (15.4)	90 (15.8)	0.824
Thromboembolic	26 (2.9)	7 (1.2)	*0.027*
Neurological (excluding complication regarding recurrent nerve)	39 (4.4)	34 (6.0)	0.176
Post-operative complications (2020-2022)	(*n* = 453)	(*n* = 284)	
Complication requiring surgical, endoscopic or radiological intervention	59 (13.0)	24 (8.5)	0.516
Complication leading to admission to ICU	44 (9.7)	16 (5.6)	

The italic values indicate statistical significance at *P* < 0.05.

CROSS, Chemoradiotherapy for Oesophageal Cancer followed by Surgery Study; ICU, intensive care unit.

a Likelihood ratio test.

Similarly, no significant difference in post-operative mortality was evident between both groups (≤30 days: O-CROSS versus E-CROSS: 1.8% versus 2.2%, respectively; ≤90 days: 3.0% versus 4.5%, *P* = 0.774, respectively).

### Undefined group

**Supplementary Fig S4:**
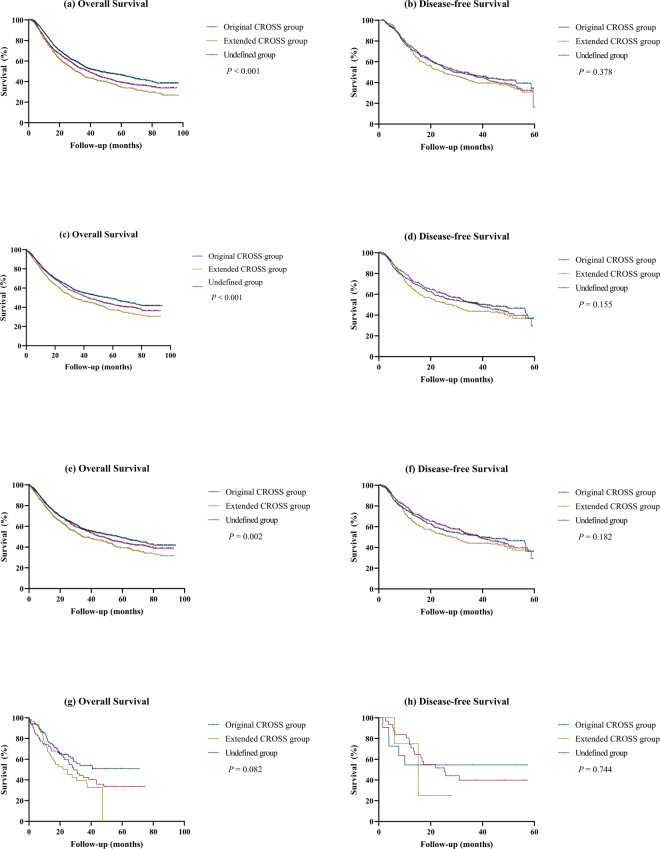
**The overall and disease-free survival in the original versusextended CROSS group versusthe undefined group.** From first date of neoadjuvant chemoradiotherapy (a,b); from date of surgery (c,d); from date of surgery performed within sixteen weeks (e,f); from date of surgery performed later than sixteen weeks (g,h).

To assess the potential impact of the loss of 1755 patients, we conducted supplementary analyses including the undefined group (Supplementary Figure S3, available at https://doi.org/10.1016/j.esmoop.2025.105098). The OS and DFS of the undefined group overlapped with those of the O-CROSS and E-CROSS groups, suggesting that the undefined group likely included patients from both O-CROSS and E-CROSS. The median OS-nCRT of the undefined group was 38.7 months ((95% CI 34.3-43.1 months, *P* < 0.001) and 43.8 months for OS-surgery (95% CI 37.8-49.8 months, *P* < 0.001). Additionally, the median DFS-nCRT of the undefined group was 32.7 months ((95% CI 27.6-37.8 months, *P* = 0.365) and 38.5 months for DFS-surgery ((95% CI 32.1-44.9 months, *P* = 0.155) (Supplementary Figure S4, available at https://doi.org/10.1016/j.esmoop.2025.105098). By inclusion of the undefined group in the OS and DFS analyses, the analyses did not change the significance of the OS and DFS compared with the OS and DFS with only the O-CROSS and E-CROSS groups (Supplementary Figure S4, Tables S6 and S7, available at https://doi.org/10.1016/j.esmoop.2025.105098).

## Discussion

In this retrospective national cohort study, the effect of extending the ‘original’ CROSS inclusion criteria was evaluated on OS, DFS, pathological response, surgical radicality, post-operative morbidity and mortality. This study showed that OS (both OS-nCRT and OS-surgery) was significantly lower in the E-CROSS group compared with the O-CROSS group. When adjusted for baseline covariates (i.e. E-CROSS criteria: age, WHO performance score, weight loss), however, there were no significant differences between O-CROSS and E-CROSS in OS-nCRT and OS-surgery. This implies that the lower OS of E-CROSS patients is caused by their overall worse prognosis on survival due to the extended criteria and not due to the CROSS regimen. Moreover, there was no difference between both groups in DFS-nCRT and DFS-surgery, pathological response, and surgical radicality. A higher number of anastomotic leakages and thromboembolic post-operative complications were observed in the O-CROSS group, yet these did not affect post-operative mortality.

The impact of applying the CROSS regimen on OS outcomes in real-world data remains contradictory. In contrast to our findings, De Heer et al.^2^ (*n* = 161) demonstrated a significant difference in OS (median 37.3 months, 95% CI 13.8-20.7 months and median 17.2 months, 95% CI 13.8-20.7 months; *P* = 0.004) in the O-CROSS and E-CROSS group, respectively. Also Wong et al.^6^ demonstrated that the E-CROSS group (*n* = 42) exhibited a significantly worse OS in ESCC patients compared with the O-CROSS group (*n* = 46); median survival 24.2 months versus 12.7 months, *P* = 0.047. Bhattacharyya et al.^7^ showed no difference in OS between the O-CROSS group and E-CROSS group in ESCC patients, which is in line with results in our study. When stratified by histopathology, our results showed a similar more favorable OS for ESCC patients compared with EAC patients treated according to the CROSS therapy than EAC, which is in line with the literature.^8^, ^9^, ^10^, ^11^ It remains challenging, however, to compare real-world data as these studies apply different extending criteria to pursue the CROSS treatment. De Heer et al.^2^ included tumor length >8 cm, >10% weight loss, tumor >2-4 cm extension in the stomach, celiac lymph node metastases, and/or age >75 years. Wong et al.^6^ used age >75 years AJCC 6th edition staging M1a/b (based on expanded lymph node criteria), or tumor length >8 cm, whereas Bhattacharyya et al.^7^ applied tumor length >8 cm, heavy nodal burden, and T4a disease. Furthermore, two updated versions of the AJCC TNM staging have been introduced since the publication of the CROSS study. In the most updated 8th edition of the AJCC TNM staging, celiac lymph nodes are included in the N staging instead of the M staging with refining of the grade differentiation and stage subgroups in pT3N0M0 ESCC. As a result, studies based on the 6th AJCC TNM edition, including that of Wong et al.^6^ and Bhattacharyya et al.^7^ are not directly comparable.

This study showed no significant difference in DFS-nCRT and DFS-surgery between the O-CROSS and E-CROSS groups. Similarly, when stratifying the results regarding the histological subtypes, no significant differences were found between the O-CROSS and E-CROSS groups either. Older patients generally have a shorter time interval until death, which seems to affect the OS and DFS estimates. Therefore, we conducted the same analyses for OS and DFS after excluding those patients in the E-CROSS group who were solely included based on stretching age >75 years (*n* = 28). We showed that age >75 years was not a dominant factor for a worse OS and DFS, as there was no clear difference in either OS (*P* = 0.321) or DFS (*P* = 0.980). Our findings on DFS align with and reinforce the results of our previous, smaller-scale study, which also did not find significant differences between both groups.^2^ These results might suggest that the CROSS therapy yields comparable outcomes for both O-CROSS and E-CROSS groups in terms of recurrence rates.

In the current study, we found that the O-CROSS group had a significant higher number of anastomotic leakage and thromboembolic complications compared with the E-CROSS group. Although it is difficult to prove, a possible explanation for the higher number of thromboembolic post-operative complications in the O-CROSS group could be that the E-CROSS patients received different perioperative care as they are generally less fit and/or older. Moreover, the significant difference in anastomotic leakages between the O-CROSS and E-CROSS groups may be explained by the higher number of cervical anastomoses. A total of 447 O-CROSS and 297 E-CROSS patients underwent a transhiatal or transthoracic McKeown procedure with a cervical anastomosis (Supplementary Table S8, available at https://doi.org/10.1016/j.esmoop.2025.105098). An additional analysis within the patient group of cervical anastomoses showed that a higher number of O-CROSS patients suffered from an anastomotic leakage compared with E-CROSS patients (21.0% versus 15.2%, *P* = 0.042). This higher number of anastomotic leakages, however, did not result in a higher mortality rate in the O-CROSS group. We should realize that esophagectomy is still a highly invasive procedure with multiple serious potential post-operative complications. Considering a switch to alternative options, i.e. definitive chemoradiotherapy or a wait-and-see procedure after nCRT in clinical complete responders remains necessary, particularly in those who are mentally or physically not fit enough at the time to undergo the surgical part of the CROSS regimen. More research in novel less invasive methods to accurately predict pCR is still desirable to refrain from unnecessary treatments.

Noteworthy are the recently published results of the ESOPEC study (*n* = 438) on improved OS of the perioperative chemotherapy combination with 5-fluorouracil, leucovorin, oxaliplatin, and docetaxel (FLOT) compared with the CROSS regimen in resectable EAC.^12^ These results of perioperative chemotherapy combination with FLOT can be accepted as a good alternative to the CROSS therapy for patients with EAC and many positive lymph nodes (cN+).

Although the current study has included as many potential confounders as possible to provide a realistic representation of OS and DFS in EC patients, it is still challenging to account for all potential confounders in a real-world data study, as not all variables observed in the real world are available in the database (i.e. socioeconomic status, smoking, alcohol consumption). Another point of discussion is that the Netherlands Cancer Registry database did not contain all data on the cause of death, which may underexpose the real attributable role in tumor-related survival.

### Conclusion

Extending the original eligibility CROSS criteria in locally advanced EC patients was associated with a lower OS. This was caused, however, by the higher age, weight loss >10%, and WHO score in the E-CROSS group. Moreover, extending the original CROSS criteria had no impact on DFS, pCR, surgical radicality, and post-operative mortality. The CROSS regimen can be used in a ‘real-world’ setting but individual factors that may contribute to OS should be considered in decision-making.
