# Supplementary material for: Impact of extending the original criteria in the Chemoradiotherapy for Oesophageal Cancer followed by Surgery Study (CROSS) regimen on treatment outcome in locally advanced esophageal cancer patients
Source: ESMO Open. 2025 May 15;10(5):105098. doi: 10.1016/j.esmoop.2025.105098 (PMC12145669; doi:10.1016/j.esmoop.2025.105098)
Supplement: Supplementary Tables [file mmc1.docx]

**Supplementary material tables**

**S1. Association of prognostic factors with OS from start nCRT from uni- and multivariable Cox regression analysis**

|  | **Univariable analysis**  **main effect** | | **Univariable analysis**  **interaction effect* CROSS subgroup** | | **Multivariable analysis**  **Main effect** | | **Multivariate analysis**  **Interaction effect*Extended CROSS** | |
| --- | --- | --- | --- | --- | --- | --- | --- | --- |
|  | **HR (95% CI)** | **P-value** | **HR (95% CI)** | **P-value** | **HR (95% CI)** | **P-value** | **HR (95% CI)** | **P-value** |
| **Sex (male)** | 1.32 (1.09-1.59) | *0.009* | 0.75 (0.57-0.98) | *0.033* | 1.15 (0.89-1.48) | 0.245 | 0.92 (0.63-1.33) | 0.641 |
| **Extended CROSS group** | 1.35 (1.03-1.50) | *<0.001* | - | - | 1.20 (0.86-1.68) | 0.286 | *-* | *-* |
| **Tumor length** | 1.03 (0.99-1.07) | 0.133 | 0.98 (0.94-1.03) | 0.379 | - | - | *-* | *-* |
| **Age** | 1.01 (1.00-1.02) | *0.021* | 1.00 (0.98-1.01) | 0.480 | 1.01 (1.00-1.02) | 0.140 | *-* | *-* |
| **WHO performance status**  0  1  2  3 (n=3) | 1.00  1.33 (1.15-1.54)  0.80 (0.44-1.46)  3.10 (0.99-9.68) | *0.001*  *<0.001*  0.473  *0.052* | 1.00  1.01 (0.81-1.26)  1.63 (0.80-3.34)  NA | 0.400  0.938  0.178  NA | 1.00  1.27 (1.09-1.46)  0.93 (0.59-1.49)  1.88 (0.26-13.74) | 0.050  *0.002*  0.768  0.532 | - | - |
| **Weight loss** | 1.02 (1.00-1.04) | 0.134 | 0.98 (0.96-1.01) | 0.154 | - | - | *-* | *-* |
| **Squamous cell carcinoma** | 0.80 (0.67-0.97) | *0.022* | 0.89 (0.67-1.19) | 0.426 | 1.11 (0.88-1.42) | 0.380 | *-* | *-* |
| **Pathologic differentiation**  High grade | 1.57 (1.34-1.83) | *<0.001* | 0.98 (0.77-1.23) | 0.831 | 1.34 (1.24-1.65) | *<0.001* | - | - |
| **Clinical TNM staging**  1B  2  2A  2B  3  3A  3B  3C  4A  X | 1.00  0.74 (0.45-1.21)  1.02 (0.65-1.61)  0.99 (0.66-1.49)  1.33 (0.92-1.91)  1.65 (1.09-2.50)  1.68 (1.05-2.69)  1.60 (0.71-3.62)  1.79 (1.20-2.66)  0.59 (0.23-1.51) | *<0.001*  0.228  0.918  0.956  0.130  *0.019*  *0.032*  0.262  *0.004*  0.267 | 1.00  1.10 (0.48-2.54)  1.81 (0.89-3.68)  1.15 (0.59-2.27)  1.24 (0.68-2.25)  0.81 (0.41-1.61)  1.15 (0.55-2.39)  2.05 (0.68-6.13)  1.02 (0.54-1.93)  1.59 (0.43-5.96) | 0.315  0.817  0.100  0.682  0.489  0.550  0.711  0.201  0.952  0.490 | 1.00  0.51 (0.28-0.93)  1.01 (0.66-1.56)  0.95 (0.64-1.42)  0.95 (0.67-1.37)  1.10 (0.73-1.65)  1.04 (0.66-1.63)  1.33 (0.67-2.64)  1.05 (0.71-1.55)  0.78 (0.37-1.63) | 0.426  *0.027*  0.951  0.817  0.793  0.640  0.870  0.418  0.815  0.507 | *-* | *-* |
| **ypT-stage**  Tx  T0  T1a  T1b  T2  T3  T4a  T4b (n=4) | 0.71 (0.22-2.24)  1.00  1.42 (0.86-2.35)  1.52 (1.12-2.06)  1.68 (1.28-2.20)  2.63 (2.08-3.33)  2.78 (0.88-8.78)  6.01 (2.20-16.44) | *<0.001*  0.556  0.172  *0.007*  *<0.001*  *<0.001*  0.081  *<0.001* | 1.76 (0.38-8.11)  1.00  0.54 (0.23-1.28)  0.85 (0.53-1.38)  0.99 (0.66-1.50)  0.74 (0.52-1.05)  2.31 (0.56-9.56)  NA | 0.191  0.468  0.161  0.518  0.967  0.090  0.247  NA | 0.20 (0.03-1.64)  1.00  2.58 (0.40-16.82)  2.75 (0.45-17.03)  3.05 (0.49-18.97)  3.06 (0.49-18.97)  2.07 (0.28-15.41)  8.17 (1.00-67.11) | 0.240  0.134  0.321  0.276  0.233  0.231  0.479  0.050 | *-* | *-* |
| **ypN-stage**  N0  N1  N2  N3 | 1.00  1.94 (1.59-2.37)  3.21 (2.54-4.07)  5.91 (4.40-7.95) | *<0.001*  *<0.001*  *<0.001*  *<0.001* | 1.00  0.98 (0.73-1.32)  0.91 (0.63-1.30)  0.94 (0.59-1.50) | 0.958  0.875  0.597  0.781 | 1.00  1.84 (1.55-2.19)  2.54 (2.05-3.15)  4.47 (3.38-5.92) | *<0.001*  *<0.001*  *<0.001*  *<0.001* | *-* | *-* |
| **Resection**  R0  R1  R2 (n=4) | 1.00  2.18 (1.68-2.84)  NA | *<0.001*  *<0.001*  NA | 1.00  0.78 (0.52-1.17)  NA | 0.473  0.224  NA | 1.00  1.28 (1.01-1.63)  11.68 (3.46-39.43) | *<0.001*  *0.043*  *<0.001* | *-* | *-* |
| **Tumor regression grade**  Complete regression  Subtotal response  Partial response  No response | 1.00  1.66 (1.27-2.17)  2.15 (1.71-2.71)  3.33 (2.34-4.73) | *<0.001*  *<0.001*  *<0.001*  *<0.001* | 1.00  0.74 (0.49-1.12)  0.91 (0.64-1.28)  1.00 (0.575-1.741) | 0.489  0.158  0.579  0.999 | 1.00  0.48 (0.08-2.97)  0.49 (0.08-3.02)  0.70 (0.11-4.39) | *0.049*  0.429  0.440  0.700 | *-* | *-* |
| **Comorbidities**  Myocardial infarction  Perivascular disease  COPD  Diabetes Mellitus  Renal disease  Mild liver disease | 1.06 (0.76-1.47)  1.29 (0.96-1.72)  0.97 (0.78-1.19)  1.13 (0.92-1.38)  1.70 (1.11-2.59)  1.34 (0.50-3.57) | 0.749  *0.089*  0.744  0.242  *0.015*  0.562 | 1.15 (0.74-1.80)  0.69 (0.44-1.08)  1.08 (0.80-1.46)  1.06 (0.80-1.41)  0.87 (0.46-1.62)  1.20 (0.37-3.90) | 0.537  0.102  0.616  0.666  0.651  0.768 | 1.04 (0.78-1.39)  -  -  1.43 (0.87-2.36)  - | 0.806  0.158 | -  -  -  -  - | *-*  *-*  *-*  *-*  *-* |
| **Post-operative complications (2015-2019)**  Chyle leak  Complication regarding n. recurrens  Wound abscess/infection  Anastomotic leakage  Pulmonary  Cardiac  Thromboembolic  Neurological (excluding complication regarding n. recurrens) | 0.95 (0.72-1.26)  0.66 (0.38-1.14)  0.86 (0.66-1.10)  1.21 (0.98-1.49)  1.09 (0.92-1.30)  0.95 (0.74-1.20)  1.30 (0.80-2.10)  1.32 (0.89-1.95) | 0.731  0.134  0.225  *0.079*  0.311  0.649  0.288  0.163 | 0.81 (0.50-1.29)  1.21 (0.51-2.85)  0.92 (0.61-1.38)  0.95 (0.67-1.33)  0.91 (0.70-1.19)  1.29 (0.86-1.75)  0.64 (0.21-1.91)  1.03 (0.59-1.80) | 0.372  0.663  0.689  0.752  0.493  0.255  0.421  0.913 | -  -  -  1.22 (1.00-1.49)  -  -  -  - | *0.049* | -  -  -  -  -  -  -  - | -  -  -  -  -  -  -  - |
| **Post-operative complications (2020-2022)**  No  Complication requiring surgical, endoscopic or radiological intervention  Complication leading to admission to ICU | 1.00  0.86 (0.49-1.50)  1.23 (0.69-2.20) | 0.660  0.589  0.477 | 1.00  1.14 (0.45-2.89)  0.92 (0.32-2.65) | 0.953  0.787  0.883 | - | - | - | - |

Abbreviations: ypT-stage = pathological T-stage, ypN-stage = pathological N-stage, R0 = microscopically radical, R1 = microscopically irradical resection, R2 = locoregional tumor residue, ICU = intensive care unit, NA = not applicable, COPD = chronic obstructive pulmonary disease

**S2. Association of prognostic factors with OS from date surgery from uni- and multivariable Cox regression analysis**

|  | **Univariate analysis**  **main effect** | | **Univariate analysis**  **interaction effect*Extended CROSS** | | **Multivariate analysis**  **Main effect** | | **Multivariate analysis**  **Interaction effect*Extended CROSS** | |
| --- | --- | --- | --- | --- | --- | --- | --- | --- |
|  | **HR (95% CI)** | **P-value** | **HR (95% CI)** | **P-value** | **HR (95% CI)** | **P-value** | **HR (95% CI)** | **P-value** |
| **Sex (male)** | 1.34 (1.07-1.67) | *0.009* | 0.81 (0.59-1.10) | 0.179 | 1.07 (0.89-1.30) | 0.472 | - | - |
| **Extended CROSS group** | 1.29 (1.14-1.46) | *<0.001* | - | - | 1.15 (0.99-1.34) | 0.070 |  |  |
| **Tumor length** | 1.01 (0.97-1.06) | 0.576 | 0.98 (0.93-1.04) | 0.550 | - | *-* | *-* | *-* |
| **Age** | 1.01 (1.00-1.02) | *0.038* | 0.99 (0.98-1.01) | 0.321 | 1.01 (1.00-1.01) | 0.191 | *-* | *-* |
| **WHO performance status**  0  1  2  3 (n=3) | 1.00  1.23 (1.04-1.45)  0.55 (0.25-1.23)  NA | *0.053*  *0.017*  0.147  NA | 1.00  1.07 (0.82-1.39)  2.63 (1.02-6.79)  NA | 0.131  0.622  *0.045*  NA | 1.00  1.25 (1.08-1.45)  0.95 (0.59-1.51)  1.83 (0.25-13.33) | *0.041*  *0.003*  0.813  0.552 |  |  |
| **Weight loss** | 1.01 (0.99-1.03) | 0.409 | 0.99 (0.97-1.01) | 0.244 | *-* | *-* | *-* | *-* |
| **Squamous cell carcinoma** | 0.79 (0.63-0.98) | *0.036* | 0.80 (0.56-1.14) | 0.222 | 1.08 (0.85-1.38) | 0.509 | - | - |
| **Pathologic differentiation**  High grade | 1.58 (1.32-1.88) | *<0.001* | 0.929 (0.710-1.217) | 0.595 | 1.44 (1.25-1.66) | *<0.001* |  |  |
| **Clinical TNM staging**  1B  2  2A  2B  3  3A  3B  3C  4A  X | 1.00  0.62 (0.35-1.11)  0.97 (0.61-1.55)  0.96 (0.62-1.47)  1.20 (0.82-1.75)  1.48 (0.95-2.29)  1.45 (0.88-2.40)  1.20 (0.46-3.08)  1.57 (1.03-2.42)  0.50 (0.17-1.41) | *0.003*  0.107  *0.905*  0.837  0.362  *0.080*  0.147  0.711  *0.038*  0.187 | 1.00  0.77 (0.27-2.23)  1.87 (0.89-3.95)  1.35 (0.66-2.77)  1.26 (0.67-2.38)  0.83 (0.40-1.72)  1.18 (0.54-2.59)  2.35 (0.67-8.22)  0.96 (0.48-1.94)  2.10 (0.52-8.55) | 0.228  0.633  0.099  0.411  0.477  0.614  0.681  0.181  0.917  0.299 | 1.00  0.55 (0.31-0.99)  1.00 (0.65-1.54)  0.94 (0.63-1.41)  1.01 (0.70-1.44)  1.14 (0.76-1.71)  1.05 (0.67-1.64)  1.31 (0.66-2.60)  1.12 (0.76-1.65)  0.83 (0.40-1.74) | 0.470  *0.048*  0.987  0.777  0.965  0.518  0.845  0.444  0.576  0.622 | *-* | *-* |
| **ypT-stage**  Tx  T0  T1a  T1b  T2  T3  T4a  T4b (n=4) | 0.53 (0.13-2.13)  1.00  1.43 (0.86-2.37)  1.52 (1.12-2.05)  1.67 (1.28-2.19)  2.64 (2.09-3.34)  2.75 (0.87-8.69)  6.09 (2.23-16.67) | *<0.001*  0.367  0.164  *0.007*  *<0.001*  *<0.001*  *0.084*  *<0.001* | 2.26 (0.37-13.87)  1.00  0.56 (0.24-1.32)  0.86 (0.53-1.39)  0.99 (0.66-1.50)  0.74 (0.52-1.05)  2.57 (0.62-10.62)  NA | 0.161  0.379  0.188  0.535  0.970  0.089  0.192  0.379 | 0.21 (0.03-1.71)  1.00  2.57 (0.38-17.27)  2.75 (0.43-17.53)  3.05 (0.47-19.58)  3.07 (0.48-19.70)  2.46 (0.32-18.75)  8.58 (1.02-72.51) | 0.225  0.144  0.331  0.286  0.241  0.236  0.386  *0.048* | *-* | *-* |
| **ypN-stage**  N0  N1  N2  N3 | 1.00  1.95 (1.59-2.38)  3.20 (2.53-4.06)  5.94 (4.42-8.00) | *<0.001*  *<0.001*  *<0.001*  *<0.001* | 1.00  0.98 (0.73-1.32)  0.91 (0.63-1.30)  0.92 (0.57-1.46) | 0.949  0.882  0.598  0.710 | 1.00  1.86 (1.56-2.20)  2.50 (2.02-3.10)  4.23 (3.19-5.60) | *<0.001*  *<0.001*  *<0.001*  *<0.001* | *-* | *-* |
| **Resection**  R0  R1  R2 (n=4) | 1.00  2.20 (1.69-2.87)  NA | *<0.001*  *<0.001*  NA | 1.00  0.77 (0.51-1.15)  NA | 0.442  0.204  NA | 1.00  1.28 (1.00-1.62)  12.36 (3.68-41.60) | *<0.001*  *0.046*  *<0.001* | *-* | *-* |
| **Tumor regression grade**  Complete regression  Subtotal response  Partial response  No response | 1.00  1.65 (1.27-2.16)  2.16 (1.72-2.72)  3.34 (2.35-4.75) | *<0.001*  *<0.001*  *<0.001*  *<0.001* | 1.00  0.74 (0.49-1.13)  0.91 (0.64-1.28)  1.02 (0.59-1.77) | 0.480  0.160  0.579  0.949 | 1.00  0.50 (0.08-3.20)  0.50 (0.08-3.16)  0.74 (0.11-4.78) | *0.028*  0.464  0.459  0.748 | *-* | *-* |
| **Comorbidities**  Myocardial infarct  Perivascular disease  COPD  Diabetes Mellitus  Renal disease  Mild liver disease | 1.06 (0.72-1.56)  1.44 (1.04-1.98)  0.89 (0.69-1.14)  1.08 (0.85-1.37)  1.56 (0.90-2.70)  1.68 (0.63-4.49) | 0.774  *0.028*  0.358  0.550  0.113  0.302 | 1.00 (0.58-1.71)  0.60 (0.35-1.02)  1.22 (0.85-1.75)  1.16 (0.83-1.63)  0.70 (0.29-1.64)  0.99 (0.28-3.51) | 0.995  *0.058*  0.282  0.377  0.406  0.982 | -  1.32 (0.93-1.87)  -  -  -  - | 0.117 | *-*  0.52 (0.28-0.97)  *-*  *-*  *-*  *-* | *0.040* |
| **Post-operative complications**  Chyle leak  Complication regarding n. recurrens  Wound abscess/infection  Anastomotic leakage  Pulmonal  Cardiac  Thromboembolic  Neurological (excluding complication regarding n. recurrens) | 1.08 (0.81-1.43)  0.73 (0.42-1.27)  0.97 (0.75-1.26)  1.41 (1.14-1.75)  1.30 (1.08-1.56)  1.08 (0.85-1.38)  1.50 (0.93-2.43)  1.51 (1.02-2.23) | 0.603  0.269  0.827  *0.002*  *0.005*  0.535  *0.099*  *0.042* | 0.84 (0.52-1.35)  1.27 (0.54-3.10)  0.96 (0.63-1.45)  0.97 (0.69-1.38)  0.95 (0.72-1.26)  1.31 (0.91-1.88)  0.64 (0.21-1.91)  1.08 (0.62-1.90) | 0.475  0.581  0.828  0.880  0.732  0.143  0.420  0.776 | -  -  -  1.12 (0.91-1.37)  1.28 (1.09-1.51)  -  1.17 (0.73-1.86)  1.31 (0.96-1.79) | 0.293  *0.003*  0.511  0.087 | - | - |
| **Post-operative complications (2020-2022)**  No  Complication requiring surgical, endoscopic or radiological intervention  Complication leading to admission to ICU | 1.00  0.87 (0.50-1.52)  1.27 (0.71-2.27) | 0.626  0.629  0.414 | 1.00  1.16 (0.46-2.94)  0.90 (0.31-2.57) | 0.933  0.759  0.838 | - | - | - | - |

Abbreviations: ypT-stage = pathological T-stage, ypN-stage = pathological N-stage, R0 = microscopically radical resection, R1 = microscopically irradical resection, R2 = locoregional tumor residue, ICU = intensive care unit, NA = not applicable

**S3. Association of prognostic factors with DFS from start nCRT from uni- and multivariable Cox regression analysis**

|  | **Univariate analysis**  **main effect** | | **Univariate analysis**  **interaction effect*Extended CROSS** | | **Multivariate analysis**  **Main effect** | | **Multivariate analysis**  **Interaction effect*Extended CROSS** | |
| --- | --- | --- | --- | --- | --- | --- | --- | --- |
|  | **HR (95% CI)** | **P-value** | **HR (95% CI)** | **P-value** | **HR (95% CI)** | **P-value** | **HR (95% CI)** | **P-value** |
| **Sex (male)** | 1.32 (0.94-1.86) | 0.113 | 0.76 (0.47-1.24) | 0.272 | - | - | - | - |
| **Extended CROSS group** | 1.14 (0.94-1.38) | 0.188 | - | - | 2.00 (0.33-12.0) | 0.449 | *-* | *-* |
| **Tumor length** | 1.06 (0.99-1.13) | 0.126 | 0.96 (0.89-1.03) | 0.252 | - | *-* | *-* | *-* |
| **Age** | 1.00 (0.98-1.02) | 0.947 | 0.98 (0.96-1.00) | *0.082* | 1.00 (0.98-1.02) | 0.911 | 0.99 (0.96-1.02) | 0.530 |
| **WHO performance status**  0  1  2  3 (n=3) | 1.00  1.11 (0.86-1.43)  1.01 (0.37-2.73)  NA | 0.602  0.441  0.985  NA | 1.00  1.15 (0.77-1.72)  1.32 (0.40-4.36)  NA | 0.748  0.503  0.645  NA | - | - | - | - |
| **Weight loss** | 1.00 (0.97-1.04) | 0.977 | 1.01 (0.97-1.05) | 0.772 | *-* | *-* | *-* | *-* |
| **Squamous cell carcinoma** | 0.79 (0.57-1.10) | 0.158 | 1.00 (0.61-1.63) | 0.995 | - | - | - | - |
| **Pathologic differentiation**  High grade | 1.49 (1.13-1.97) | *0.005* | 1.09 (0.72-1.66) | 0.690 | 1.49 (1.19-1.87) | *<0.001* | - | - |
| **Clinical TNM staging**  1B  2  2A  2B  3  3A  3B  3C  4A  X | 1.00  0.62 (0.24-1.57)  0.70 (0.42-1.16)  0.76 (0.45-1.28)  1.38 (0.88-2.17)  1.43 (0.92-2.23)  1.66 (1.01-2.73)  1.81 (0.70-4.66)  1.43 (0.72-2.85)  0.63 (0.15-2.63) | *0.005*  0.302  0.165  0.301  0.167  0.116  *0.046*  0.220  0.313  0.525 | 1.00  1.15 (0.24-5.61)  2.03 (0.86-4.80)  2.25 (0.94-5.40)  1.21 (0.55-2.68)  1.06 (0.48-2.36)  1.22 (0.53-2.84)  2.53 (0.72-8.86)  1.36 (0.46-4.04)  5.53 (0.94-32.54) | 0.228  0.859  0.107  *0.068*  0.639  0.880  0.640  0.146  0.578  *0.058* | 1.00  0.73 (0.30-1.80)  0.71 (0.44-1.16)  0.89 (0.55-1.45)  1.03 (0.66-1.59)  1.21 (0.78-1.87)  0.95 (0.58-1.55)  1.83 (0.88-3.83)  1.04 (0.53-2.05)  1.07 (0.44-2.58) | 0.307  0.497  0.173  0.648  0.910  0.401  0.834  0.108  0.913  0.882 | *-* | *-* |
| **ypN-stage**  N0  N1  N2  N3 | 1.00  2.39 (1.73-3.29)  3.53 (2.39-5.21)  5.81 (3.50-9.65) | *<0.001*  *<0.001*  *<0.001*  *<0.001* | 1.00  0.80 (0.49-1.31)  1.04 (0.59-1.84)  0.64 (0.28-1.43) | 0.585  0.375  0.897  0.277 | 1.00  2.11 (1.61-2.78)  2.66 (1.91-3.69)  3.39 (2.13-5.39) | *<0.001*  *<0.001*  *<0.001*  *<0.001* | *-* | *-* |
| **Resection**  R1 | 2.82 (1.71-4.64) | *<0.001* | 0.62 (0.29-1.33) | 0.220 | 1.03 (0.91-1.02) | 0.652 | *-* | *-* |
| **Tumor regression grade**  Complete regression  Subtotal response  Partial response  No response | 1.00  1.67 (1.06-2.62)  2.81 (1.90-4.15)  5.79 (3.39-9.88) | *<0.001*  *0.026*  *<0.001*  *<0.001* | 1.00  0.86 (0.44-1.70)  0.70 (0.39-1.26)  0.63 (0.27-1.48) | 0.580  0.662  0.232  0.292 | 1.00  1.62 (1.08-2.44)  2.04 (1.41-2.93)  3.86 (2.40-6.21) | *<0.001*  *0.020*  *<0.001*  *<0.001* | *-* | *-* |

Abbreviations: R1 = microscopically irradical resection, NA = not applicable

**S4. Association of prognostic factors with DFS from date surgery from uni- and multivariable Cox regression analysis**

|  | **Univariate analysis**  **main effect** | | **Univariate analysis**  **interaction effect*Extended CROSS** | | **Multivariate analysis**  **Main effect** | | **Multivariate analysis**  **Interaction effect*Extended CROSS** | |
| --- | --- | --- | --- | --- | --- | --- | --- | --- |
|  | **HR (95% CI)** | **P-value** | **HR (95% CI)** | **P-value** | **HR (95% CI)** | **P-value** | **HR (95% CI)** | **P-value** |
| **Sex (male)** | 1.28 (0.90-1.82) | 0.174 | 0.99 (0.59-1.67) | 0.973 | - | - | - | - |
| **Extended CROSS group** | 1.18 (0.96-1.45) | 0.116 | - | - | 0.58 (0.25-1.35) | 0.206 | *-* | *-* |
| **Tumor length** | 1.07 (0.99-1.15) | *0.083* | 0.93 (0.86-1.02) | 0.111 | 0.97 (0.93-1.02) | 0.216 | *-* | *-* |
| **Age** | 1.00 (0.98-1.02) | 0.904 | 0.98 (0.96-1.01) | 0.113 | - | - | - | - |
| **WHO performance status**  0  1  2  3 (n=3) | 1.00  1.14 (0.86-1.50)  0.74 (0.18-2.98)  NA | 0.721  0.361  0.668  NA | 1.00  0.94 (0.61-1.46)  2.06 (0.43-9.97)  NA | 0.623  0.796  0.368  NA | - | - | - | - |
| **Weight loss** | 0.99 (0.96-1.03) | 0.742 | 1.01 (0.97-1.05) | 0.708 | *-* | *-* | *-* | *-* |
| **Squamous cell carcinoma** | 0.76 (0.53-1.09) | 0.130 | 0.81 (0.46-1.44) | 0.471 | - | - | - | - |
| **Pathologic differentiation**  High grade | 1.40 (1.05-1.87) | *0.024* | 1.20 (0.77-1.88) | 0.416 | 1.42 (1.12-1.79) | *0.003* | - | - |
| **Clinical TNM staging**  1B  2  2A  2B  3  3A  3B  3C  4A  X | 1.00  0.62 (0.24-1.60)  0.66 (0.38-1.14)  0.64 (0.36-1.12)  1.27 (0.79-2.04)  1.45 (0.90-2.32)  1.65 (0.97-2.80)  0.89 (0.27-2.93)  0.84 (0.37-1.93)  0.53 (0.13-2.23) | *0.002*  0.322  0.133  0.117  0.331  0.123  *0.064*  0.843  0.683  0.387 | 1.00  1.03 (0.17-6.21)  2.21 (0.87-5.63)  2.97 (1.16-7.57)  1.38 (0.59-3.22)  1.14 (0.48-2.71)  1.17 (0.46-2.95)  5.90 (1.32-26.35)  1.77 (0.48-6.55)  5.232 (0.87-31.45) | *0.055*  0.972  *0.095*  *0.023*  0.463  0.759  0.743  *0.020*  0.394  *0.071* | 1.00  0.60 (0.21-1.77)  0.51 (0.28-0.94)  0.47 (0.25-0.88)  0.74 (0.44-1.25)  1.15 (0.68-1.92)  0.77 (0.42-1.40)  0.46 (0.14-1.54)  0.58 (0.25-1.38)  0.36 (0.08-1.53) | *0.037*  0.355  *0.031*  *0.018*  0.263  0.610  0.391  0.207  0.220  0.166 | 1.00  2.58 (0.39-17.3)  2.29 (0.74-7.01)  3.53 (1.20-10.36)  2.19 (0.83-5.80)  1.41 (0.51-3.85)  2.10 (0.71-6.20)  9.74 (1.87-50.75)  2.24 (0.55-9.20)  2.19 (0.17-28.14) | 0.207  0.329  0.149  *0.022*  0.114  0.506  0.181  *0.007*  0.263  0.548 |
| **ypN-stage**  N0  N1  N2  N3 | 1.00  2.27 (1.66-3.12)  3.70 (2.51-5.46)  5.95 (3.58-9.88) | *<0.001*  *<0.001*  *<0.001*  *<0.001* | 1.00  0.87 (0.53-1.42)  1.03 (0.58-1.82)  0.66 (0.29-1.47) | 0.720  0.571  0.928  0.306 | 1.00  1.92 (1.45-2.54)  2.63 (1.88-3.68)  3.50 (2.14-5.72) | *<0.001*  *<0.001*  *<0.001*  *<0.001* | *-* | *-* |
| **Resection**  R1 | 2.57 (1.56-4.22) | *<0.001* | 0.72 (0.34-1.53) | 0.393 | 1.07 (0.93-1.23) | 0.330 | *-* | *-* |
| **Tumor regression grade**  Complete regression  Subtotal response  Partial response  No response | 1.00  1.69 (1.08-2.65)  2.76 (1.87-4.08)  5.44 (3.19-9.27) | *<0.001*  *0.023*  *<0.001*  *<0.001* | 1.00  0.79 (0.40-1.56)  0.76 (0.43-1.36)  0.71 (0.30-1.64) | 0.800  0.503  0.356  0.418 | 1.00  1.74 (1.13-2.66)  2.44 (1.67-3.57)  4.31 (2.64-7.04) | *<0.001*  *0.012*  *<0.001*  *<0.001* | *-* | *-* |

Abbreviations: R1 = microscopically irradical resection, NA = not applicable

**S5. Separate histopathological analysis of esophageal adenocarcinoma and esophageal squamous cell carcinoma**

|  | **Esophageal adenocarcinoma (EAC)** | | **P-value^a^** |
| --- | --- | --- | --- |
|  | O-CROSS (*n*=1102), *n(%)* | E-CROSS (*n*=706), *n*(%) |  |
| pCR (ypT0N0) | 212 (19.2%) | 112 (15.9%) | 0.279 |
| pCR (ypT0) | 224 (20.3%) | 133 (18.9%) | *0.006* |
| Resection  R0  R1  R2  Missing | 995 (90.3%)  92 (8.3%)  1 (0.1%)  14 (1.3%) | 622 (88.1%)  72 (10.2%)  1 (0.1%)  11 (1.6%) | 0.580 |
| Post-operative complications (2015-2019)  Chyle leak  Complication regarding recurrent nerve  Wound abscess/infection  Anastomotic leakage  Pulmonal  Cardiac  Thromboembolic  Neurological (excluding complication regarding recurrent nerve)  Post-operative complications (2020-2022)  Complication requiring surgical, endoscopic or radiological intervention  Complication leading to admission to ICU | (*n*=717)    72 (10.0%)  23 (3.2%)  107 (14.9%)  182 (25.4%)  230 (32.1%)  104 (14.5%)  21 (2.9%)  29 (4.0%)  (*n*=385)  48 (12.5%)  31 (8.1%) | (*n*=456)  35 (7.7%)  10 (2.2%)  50 (11.0%)  88 (19.3%)  149 (32.7%)  73 (16.0%)  7 (1.5%)  30 (6.6%)  (*n*=250)  20 (8.0%)  15 (6.0%) | 0.166  0.298  0.050  *0.017*  0.831  0.484  0.116  0.056  0.854 |
| Post-operative mortality  <30 days  <90 days | 16 (1.5%)  34 (3.1%) | 17 (2.4%)  35 (5.0%) | 0.940 |
|  | **Esophageal squamous cell carcinoma (ESCC)** | | P-value^a^ |
|  | O-CROSS (*n*=240), *n(%)* | E-CROSS (*n*=146), *n*(%) |  |
| pCR (ypT0N0) | 104 (43.4%) | 49 (33.6%) | 0.075 |
| pCR (ypT0) | 126 (52.5%) | 63 (43.2%) | 0.092 |
| Resection  R0  R1  R2  Missing | 221 (92.1%)  17 (7.1%)  0 (0.0%)  2 (0.8%) | 132 (90.4%)  7 (4.8%)  2 (1.4%)  5 (3.4%) | *0.047* |
| Post-operative complications (2015-2019)  Chyle leak  Complication regarding recurrent nerve  Wound abscess/infection  Anastomotic leakage  Pulmonal  Cardiac  Thromboembolic  Neurological (excluding complication regarding recurrent nerve)  Post-operative complications (2020-2022)  Complication requiring surgical, endoscopic or radiological intervention  Complication leading to admission to ICU | (*n*=172)  23 (13.4%)  7 (4.1%)  26 (15.1%)  36 (20.9%)  60 (34.9%)  33 (19.2%)  5 (2.9%)  10 (5.8%)  (*n*=68)  11 (16.2%)  13 (19.1%) | (*n*=112)  13 (11.6%)  6 (5.4%)  17 (15.2%)  20 (17.9%)  36 (32.1%)  17 (15.2%)  0 (0.0%)  4 (3.6%)  (*n*=34)  4 (11.8%)  1 (2.9%) | 0.661  0.615  0.989  0.724  0.633  0.383  *0.024*  0.384  0.066 |
| Post-operative mortality  <30 days  <90 days | 8 (3.3%)  6 (2.5%) | 2 (1.4%)  3 (2.1%) | 0.509 |

Abbreviations: pCR= pathologic complete response, ypT0N0 = pathologic complete response, ypT0 = pathologic complete local response, R0 = microscopically radical resection, R1 = microscopically irradical resection margin, R2 = locoregional tumor residue

^a^Likelihood ratio test

**S6. Patient and tumor characteristics of the complete original, extended and undefined CROSS groups**

|  | **Original CROSS (*n*=1652), *n*(%)** | **Extended CROSS (*n*=1091), *n*(%)** | **Undefined group (n=1755), n(%)** | ***P*-value^a^** |
| --- | --- | --- | --- | --- |
| Sex (male) | 1313 (79.5%) | 828 (75.9%) | 1082 (61.7%) | *0.038* |
| Age (years) (median, IQR) | 66 (60-71) | 69 (61-76) | 67 (61-72) | *<0.001* |
| Histology  Adenocarcinoma  Squamous cell carcinoma | 1324 (80.1%)  328 (19.9%) | 897 (82.2%)  194 (17.8%) | 1137 (64.8%)  281 (16.0%) | 0.331 |
| Tumor location  Proximal  Middle  Distal  Overlapping  Not specified | 11 (0.7%)  183 (11.1%)  1433 (86.7%)  8 (0.5%)  17 (1.0%) | 6 (0.5%)  131 (12.0%)  932 (85.4%)  13 (1.2%)  9 (0.8%) | 12 (0.7%)  177 (10.1%)  1202 (68.5%)  9 (0.5%)  18 (1.1%) | 0.424 |
| WHO performance score  0  1  2  3  4  Unknown  Missing | 1008 (61.0%)  612 (37.0%)  32 (1.9%)  0 (0.0%)  0 (0.0%)  0 (0.0%)  0 (0.0%) | 509 (46.7%)  477 (43.7%)  47 (4.3%)  3 (0.3%)  1 (0.1%)  54 (4.9%)  0 (0.0%) | 646 (36.8%)  498 (28.4%)  40 (2.3%)  0 (0.0%)  0 (0.0%)  223 (12.7%)  348 (19.8%) | *<0.001* |
| Tumor length (cm) (median, IQR)  Missing | 4.0 (3.0-6.0)  0 (0.0%) | 6.0 (4.0-9.0)  88 (8.1%) | 4.0 (3.0-6.0)  488 (27.8%) | *<0.001* |
| Clinical TNM stadium  1  1A  1B  2  2A  2B  3  3A  3B  3C  4A  X | 4 (0.2%)  1 (0.1%)  60 (3.6%)  116 (7.0%)  84 (5.1%)  209 (12.7%)  789 (47.8%)  103 (6.2%)  54 (3.3%)  11 (0.7%)  202 (12.2%)  19 (1.2%) | 2 (0.2%)  0 (0.0%)  33 (3.0%)  47 (4.3%)  57 (5.2%)  102 (9.3%)  521 (47.8%)  71 (6.5%)  51 (4.7%)  13 (1.2%)  179 (16.4%)  15 (1.4%) | 1 (0.1%)  0 (0.0%)  97 (5.5%)  88 (5.0%)  111 (6.3%)  205 (11.7%)  526 (30.0%)  140 (8.0%)  58 (3.3%)  17 (1.0%)  160 (9.1%)  15 (0.9%) | *<0.001* |
| **Comorbidities**  Myocardial infarct  Perivascular disease  COPD  Diabetes Mellitus  Renal disease  Mild liver disease  Ulcer disease | 85 (5.1%)  94 (5.7%)  221 (13.4%)  229 (13.9%)  32 (1.9%)  7 (0.4%)  58 (3.5%) | 79 (7.2%)  71 (6.5%)  167 (15.3%)  190 (17.4%)  28 (2.6%)  12 (1.1%)  30 (2.7%) | 71 (4.0%)  95 (5.4%)  190 (10.8%)  215 (12.3%)  18 (1.0%)  18 (1.0%)  56 (3.2%) | *0.040*  0.422  0.330  *0.042*  0.064  *0.021*  0.225 |
| Follow-up (months) (median, IQR) | 26.8 (10.7-45.0) | 21.7 (12.4-42.8) | 22.3 (9.9-52.5) | *<0.001* |

Abbreviations: IQR = interquartile range, prox = proximal, mid = middle

^a^ Likelihood ratio test
^*^ One-way Anova

**S7. Pathological tumor characteristics in the original, extended and undefined CROSS groups after curative resection**

|  | **Original CROSS (*n*=1342), *n*(%)** | **Extended CROSS (*n*=852), *n*(%)** | **Undefined group (n=1073)** | ***P*-value^a^** |
| --- | --- | --- | --- | --- |
| pCR (ypT0N0) | 316 (23.5%) | 161 (18.9%) | 225 (21.0%) | *0.024* |
| pCR (ypT0) | 353 (26.4%) | 198 (23.2%) | 263 (24.5%) | 0.137 |
| ypT-stage  Tis  T0  T1a  T1b  T2  T3  T4a  T4b  Tx | 1 (0.1%)  350 (26.1%)  48 (3.6%)  192 (14.3%)  269 (20.0%)  465 (34.6%)  5 (0.4%)  0 (0.0%)  12 (0.9%) | 0 (0.0%)  196 (23.0%)  28 (3.3%)  95 (11.2%)  157 (18.4%)  355 (41.7%)  9 (1.1%)  4 (0.5%)  7 (0.8%) | 3 (0.3%)  263 (24.5%)  33 (3.1%)  176 (16.4%)  203 (18.9%)  375 (34.9%)  8 (0.7%)  2 (0.2%)  9 (0.8%) | *0.003* |
| ypN-stage  N0  N1  N2  N3  Missing | 844 (62.9%)  295 (22.0%)  135 (10.1%)  63 (4.7%)  5 (0.4%) | 497 (58.3%)  210 (24.6%)  98 (11.5%)  42 (4.9%)  5 (0.6%) | 643 (59.9%)  242 (22.6%)  119 (11.1%)  61 (5.7%)  8 (0.7%) | 0.429 |
| Pathologic differentiation  Well  Moderate  Poorly  Unknown | 44 (4.4%)  641 (47.8%)  455 (33.9%)  202 (15.1%) | 19 (2.2%)  387 (45.4%)  323 (37.9%)  123 (14.4%) | 30 (2.8%)  496 (46.2%)  375 (34.9%)  172 (16.0%) | 0.426 |
| Resection  R0  R1  R2  Unknown  Missing | 1216 (90.6%)  109 (8.1%)  1 (0.1%)  14 (1.0%)  2 (0.2%) | 754 (88.5%)  79 (9.3%)  3 (0.4%)  12 (1.4%)  4 (0.5%) | 953 (88.8%)  70 (6.5%)  1 (0.1%)  33 (3.1%)  16 (1.5%) | *0.002* |

Abbreviations: pCR = pathologic complete response, ypT0N0 = pathologic complete response, ypT = pathologic tumor stage, ypN = pathologic node stage, R0 = microscopically radical resection, R1 = microscopically irradical resection margin, R2 = locoregional tumor residue

^a^ Likelihood ratio test

**S8. Type of surgery in the original and extended CROSS groups**

|  | **Original CROSS (*n*=1342), *n* (%)** | **Extended CROSS (*n*=852), *n* (%)** | ***P*-value^a^** |
| --- | --- | --- | --- |
| **Type of esophageal resection**  Transhiatal  Transthoracic Ivor Lewis  Transthoracic Mckeown  Transthoracic other/not specified  Minimally invasive cervical esophagectomy  Total stomach resection  Resection other/not specified | 104 (7.7%)  869 (64.8%)  343 (25.6%)  14 (1.0%)  10 (0.7%)  0 (0.0%)  2 (0.1%) | 89 (10.4%)  525 (61.6%)  208 (24.4%)  12 (1.4%)  11 (1.3%)  4 (0.5%)  3 (0.4%) | *0.014* |
| **Surgical technique**  Open surgery  Conventional scopical, no conversion  Conventional scopical, with conversion  Robot-assisted, no conversion  Robot-assisted, with conversion  Unknown  Missing | 97 (7.2%)  894 (66.6%)  26 (1.9%)  300 (22.4%)  4 (0.3%)  10 (0.7%)  11 (0.8%) | 77 (9.0%)  559 (65.6%)  22 (2.6%)  180 (21.1%)  1 (0.1%)  3 (0.4%)  10 (1.2%) | 0.325 |
| **Scopical approach**  Open  Minimal invasive abdomen  Minimal invasive thoracic  Minimal invasive abdomen and thoracic  Unknown  Missing | 86 (6.4%)  99 (7.4%)  34 (2.5%)  1104 (82.3%)  17 (1.3%)  2 (0.1%) | 63 (7.4%)  78 (9.2%)  16 (1.9%)  681 (79.9%)  10 (1.2%)  4 (0.5%) | 0.387 |

^a^ Likelihood ratio test
