# Supplementary figures and images for: Impact of extending the original criteria in the Chemoradiotherapy for Oesophageal Cancer followed by Surgery Study (CROSS) regimen on treatment outcome in locally advanced esophageal cancer patients
Source: ESMO Open. 2025 May 15;10(5):105098. doi: 10.1016/j.esmoop.2025.105098 (PMC12145669; doi:10.1016/j.esmoop.2025.105098)

(a) OS-nCRT

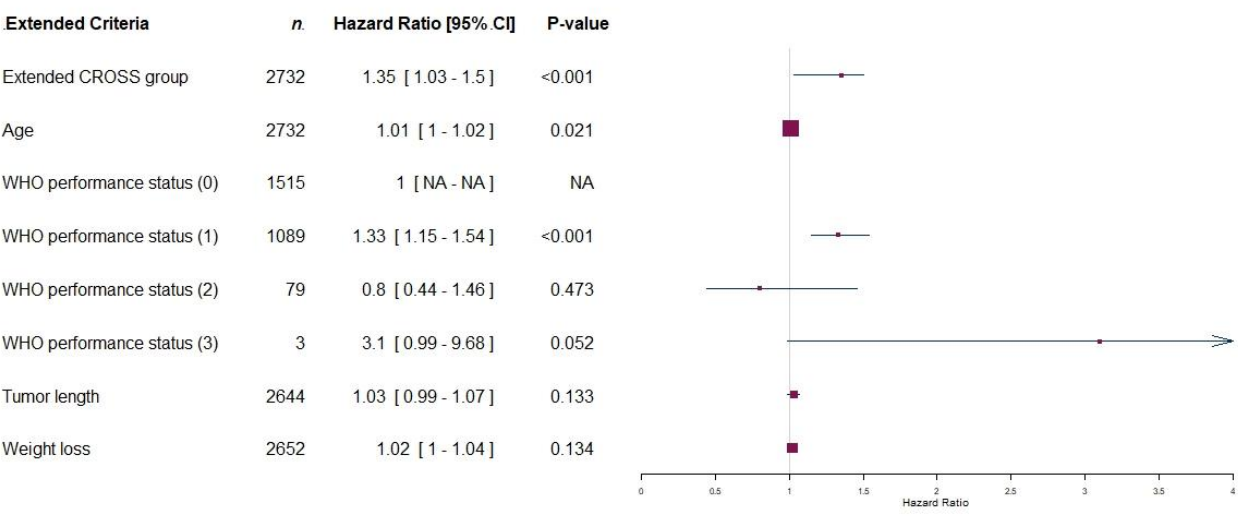

(b) DFS-nCRT

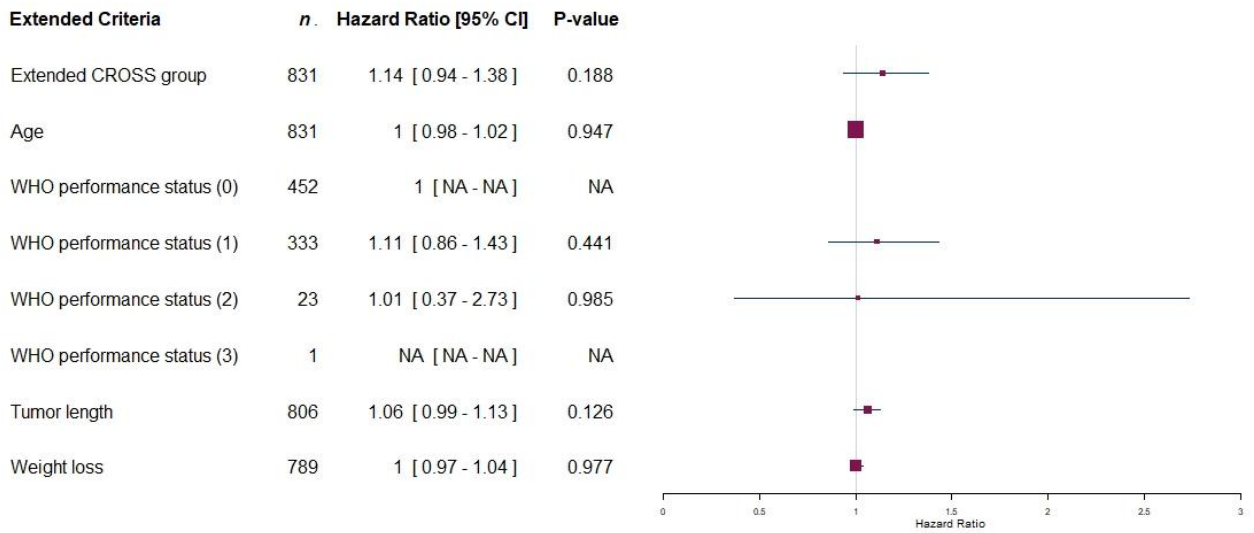

(c) OS-Surgery

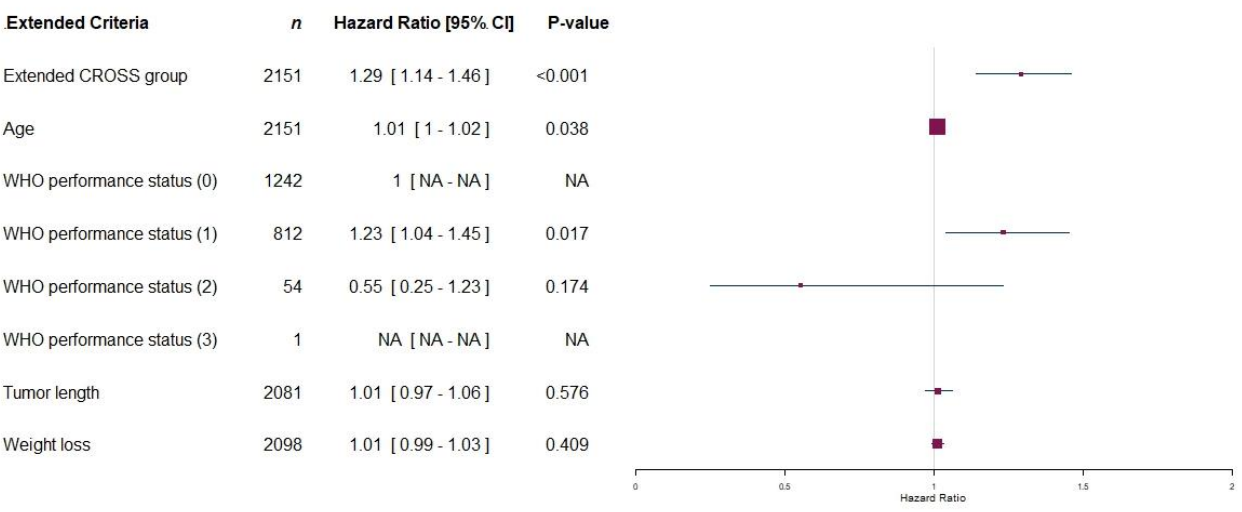

(d) DFS-surgery

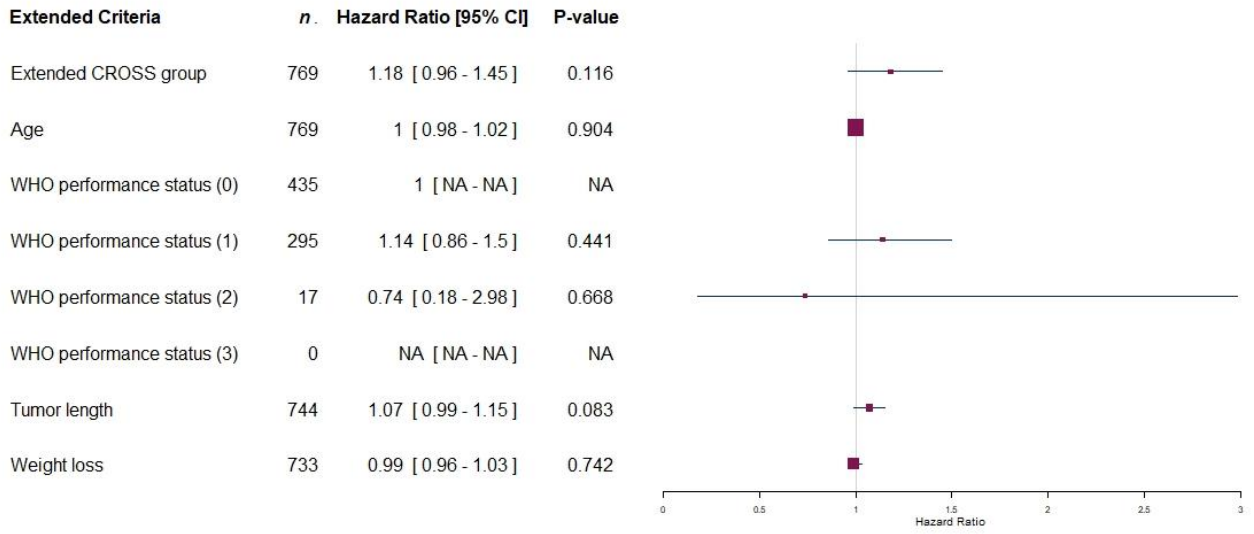

Supplement: Supplementary Fig S2 [file mmc2.pdf]

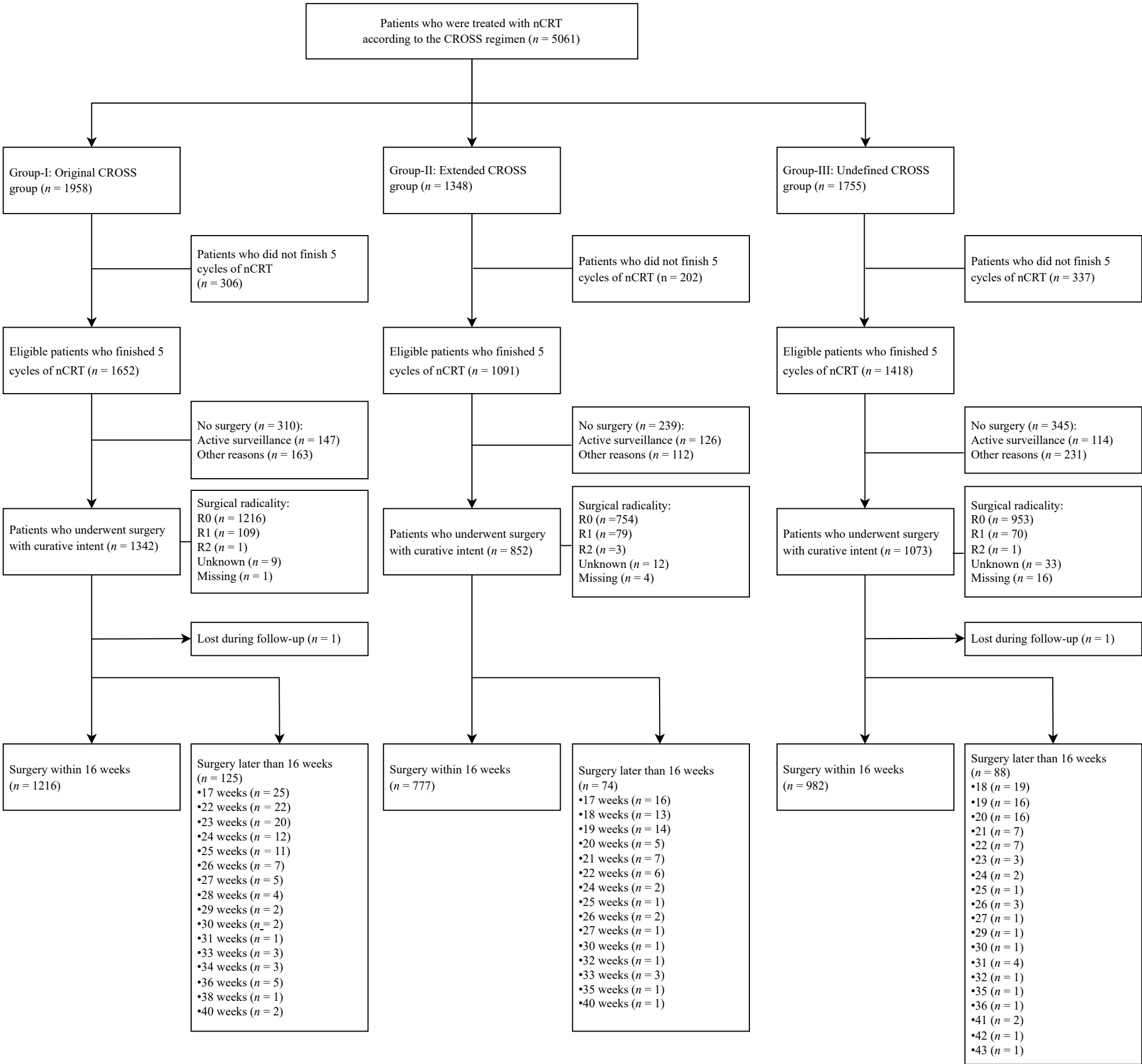

Supplement: Supplementary Fig S3 [file mmc3.pdf]
