# Supplementary material for: Impact of extending the original criteria in the Chemoradiotherapy for Oesophageal Cancer followed by Surgery Study (CROSS) regimen on treatment outcome in locally advanced esophageal cancer patients
Source: ESMO Open. 2025 May 15;10(5):105098. doi: 10.1016/j.esmoop.2025.105098 (PMC12145669; doi:10.1016/j.esmoop.2025.105098)
Supplement: Supplementary Data [file mmc4.docx]

**Figure S1. Surgical subanalyses of patients who underwent surgery, within sixteen weeks and beyond sixteen weeks**, overall survival (a,b); and disease-free survival (c,d) in the original vs. extended CROSS group

**Figure S2. Forest plots depicting the effect of individual CROSS criteria**, overall survival (a,c); and disease-free survival (b,d)

**Figure S3.** CONSORT flow diagram of patients included in the original vs. extended CROSS group vs. undefined group

**Figure S4.** The overall and disease-free survival in the original vs. extended CROSS group vs. the undefined group, from first date of neoadjuvant chemoradiotherapy (a,b); from date of surgery (c,d); from date of surgery performed within 16 weeks after nCRT (e,f); from date of surgery performed later than 16 weeks after nCRT (g,h)

Preferred magnification factor: full page width +-190mm

Supplementary Fig S1, S2 and S4 in color
